# On Acute Idiopathic Carditis

**Published:** 1829-07-01

**Authors:** 


### XLIII.

### Acute Idiopathic Carditis.

The American Journal of the Medical Sciences quotes some passages from Hecker's Annals of Medicine, for September last, a journal which we have not seen. Among these passages is one containing what is laid down as the true pathognomonic character of idiopathic carditis, by Drs. Krause and Heim of Berlin. These gentlemen consider the disease as extremely rare—and so it may be, if we restrict carditis to inflammation of the heart alone, and without any preceding inflammation in another structure, as acute rheumatism, &c. The form of the disease, too, which is described by these authors, is certainly not very common. The following is their description.

"The disease begins with shivering and trembling of the whole body, and intermittent chill, which latter when present may continue during twenty-four hours, but is followed by very little or no heat. There is no acute stitch felt, but pains in the heart precede the distinct attack for twenty-four hours. Sometimes the disease comes on suddenly. The patient has no cough ; if in any instance cough occurs it is entirely dry, neither mucus nor blood being expectorated. In the commencement the patient has the greatest anguish and the most agonizing pain, not in the chest generally, but immediately in the heart itself. The patient shrieks out and is not quiet more than a second, repeating with great force the same word three or four times to express his most distressing sensations. He does not lie still an instant, but tosses himself about in bed like one half distracted, while his arms and head are in continued motion. The patient presses upon the region of the heart, and if pressure be made by a bystander, he impetuously demands that it shall be increased. The countenance is always extremely pale. The chest is elevated, and the head, which is in perpetual motion, is thrown more backwards. The face and hands are quite cold. Before each necessary bleeding, the pulse is throughout not to be felt. The patient feels every movement of the heart, and even complains of its painful throbbing, yet when the physician applies his hand to the chest, he cannot discover the least irregularity. The stronger the pulsation of the heart is, the greater is the pain felt in the organ ; it seems to the patient to strike upon a wounded spot. He is nauseated, but does not vomit; the greater his thirst is, so much the more does he refuse to drink, even when a glass of water is held to his mouth. He is very loquacious, even when otherwise naturally silent; we might also say physically, what the Scriptures assert morally, that "out of the fulness of the heart the mouth speaketh.' Fainting and delirium are not uncommon.

"A copious bleeding is followed by so great an alleviation of anguish and pain, which lasts for several hours, that the patient thinks himself quite cured. But the symptoms suddenly return again after the cessation. The anguish and pain is increased by warm applications to the chest, and the patient throws them off. If the treatment be neglected, or improper, the patient dies either in consequence of polypous formations on the internal or external surface of the heart, as well as on the internal surface of the pericardium, or with adhesions of the heart to this membrane, and effusions of pus or water into the cavity of the pericardium.

"The case related by Dr. Krause, in which also he had the assistance of Dr. Heim, manifested the foregoing symptoms in a striking manner. By free and ample bleeding, and the use of digitalis, &c. the patient was quite restored to his usual health in ten or twelve days."

---

